# HPV type-specific prevalence using a urine assay in unvaccinated male and female 11- to 18-year olds in Scotland

**DOI:** 10.1038/bjc.2011.30

**Published:** 2011-02-22

**Authors:** M C O'Leary, K Sinka, C Robertson, K Cuschieri, R Lyman, M Lacey, A Potts, H A Cubie, M Donaghy

**Affiliations:** 1 Health Protection Scotland, Clifton House, Clifton Place, Glasgow G3 7LN, UK; 2 Department of Mathematics and Statistics, University of Strathclyde, 26 Richmond Street, Glasgow G1 1XH, UK; 3 Scottish HPV Reference Laboratory, Specialist Virology Centre, Royal Infirmary of Edinburgh, Edinburgh EH16 4SA, UK

**Keywords:** HPV prevalence, unvaccinated adolescents, Scotland

## Abstract

**Background:**

We conducted a baseline prevalence survey of unvaccinated 11- to 18-year olds to inform effectiveness studies for the new human papillomavirus (HPV) immunisation programme in Scotland.

**Methods:**

Participants were recruited from schools and colleges and invited to provide demographic data and an anonymous urine sample for type-specific HPV testing.

**Results:**

Among females aged 11–14 years, the weighted prevalence was 1.1% overall; 0.9% for high-risk types and no infections were associated with types 16 and 18. Among 15- to 18-year old females, the weighted prevalence was 15.2% overall; 12.6% for high-risk types and 6.5% for types 16 and 18. Among females aged 16–18 years, infection was more frequently associated with attending college and rural schools, and showed a trend towards increasing prevalence with increasing social deprivation (*P*=0.045). Among males aged 11–14 years, the weighted prevalence was 1.4% overall; 1.0% for high-risk types and 0.7% for types 16 and 18. Among 15- to 18-year old males, the weighted prevalence was 3.9% overall; 2.4% for high-risk types and 0.7% for types 16 and 18.

**Conclusions:**

Human Papillomavirus prevalence is low among 11- to 14-year olds, which includes the age group targeted for routine vaccination. The prevalence in males and correlation with deprivation require further investigation.

Persistent infection with high-risk types of human papillomavirus (HPV) is recognised as the principal cause of cervical cancer, the second most common cancer among women globally (Clifford *et al*, 2006). Vaccines that protect against HPV-16 and -18, the types implicated in over 70% of cervical cancers, have recently been developed (Clifford *et al*, 2006). As these vaccines are incorporated into routine immunisation programmes, it will be necessary to monitor their impact and effectiveness through routine surveillance of vaccinated cohorts. Suitable surveillance end points include changes to the incidence and prevalence of both HPV-related cervical disease (abnormal cervical smears, pre-cancerous lesions and invasive cancers) and type-specific HPV prevalence (European Centre for Disease Prevention and Control, 2007). Currently, in the United Kingdom, HPV infection is not routinely diagnosed in the clinical setting, as it is largely asymptomatic and no treatment is available. Consequently routinely collected prevalence data are usually unavailable. In particular, there is a paucity of data on the prevalence of infection among the age groups targeted for vaccination.

In Scotland, the bivalent HPV vaccine, which protects against the high-risk HPV types 16 and 18, was introduced into the national immunisation schedule in September 2008, targeting females aged 12–13 years. To facilitate the ongoing monitoring of HPV infection in vaccinated cohorts, before the introduction of the immunisation programme, we undertook a comparative baseline cross-sectional survey of unvaccinated Scottish adolescents to measure the type-specific prevalence of HPV infection and its association with age, sex and social deprivation. We report here the findings of this survey, which we believe is the first population-based prevalence survey of HPV infection among adolescent males and females in the United Kingdom and one of only a few surveys (Andersson-Ellstrom *et al*, 1996; De Sanjose *et al*, 2003; Dunne *et al*, 2007) that have included younger adolescents in the age group targeted for vaccination.

## Materials and methods

### Study design and recruitment of subjects

This study was funded by the Scottish Government and received approval from the South Glasgow and Clyde Research Ethics Committee. Between January and May 2008, we recruited males and females (11–18 years) attending publicly funded (state) and private fee-paying (independent) schools. Approximately 35% of young people in Scotland leave school by the age of 16 years (HM Inspectorate of Education, 2006), and leaving school early is correlated with social deprivation. Although we did not specifically sample early school leavers, in order to increase the representativeness of our sample, we conducted additional recruitment of those who left school early to attend colleges of further education (hereafter known as colleges).

We sampled education authorities (EAs) using population proportional to size and, within them, we invited all state and independent schools and colleges to participate. The target sample was stratified by age, with oversampling in older age groups. We aimed to recruit a sample size that would provide sufficient power to estimate the overall prevalence of HPV infection, the prevalence by age, sex and social deprivation and the specific genotypes in circulation. For state schools, we collected data on the denominational (religious) affiliation of the school and whether it was in an urban or rural setting. The achieved sample had a precision of ±3% on the estimated prevalence of HPV and a power of 80% to detect differences in prevalence of at least 8% between the two subgroups with 1000 participants.

Participants gave written informed consent, as did the parents of students aged <16 years. All the students within a selected year were invited to a presentation given by the study team, in which they were asked to take part in the study by providing an anonymous specimen of urine for HPV testing along with information on their age, sex and postal code using a standardised data-collection form. College students were recruited directly on campus on the designated study days. Samples and data forms were labelled and linked with a unique anonymous study number. Participants were not given their results. A urine sample was chosen, as it is a non-invasive method to access the population of interest.

### Laboratory analysis

As urine contains relatively few cells, an HPV assay (HPV Inno-LiPA HPV Genotyping Extra assay, Innogenetics, Gent, Belgium) with a high analytical sensitivity was used (Appendix 1). This assay was previously validated in urine, in comparison to a gold standard sample (cervical liquid-based cytology sample or penile swab), by measuring the HPV concordance by sample type (Cuschieri *et al*, 2008). Briefly, samples were collected from females and males aged <25 years attending a drop-in clinic for integrated sexual health services. The sensitivity of the assay for detection of all HPV types was 90.5% (95% confidence interval (95% CI): 79.3–96.9) for females and 55.9% (95% CI: 37.8–72.8) for males, and the specificity was 71% (95% CI: 61.0–79.5) for females and 63.2% (95% CI: 54.2–71.4) for males. Discrepancy analysis indicated that the distribution of HPV types in urine was similar to that observed in the gold standard sample for both males and females.

### Data analyses

Data were entered into a Microsoft Access database. Postal codes were automatically assigned a point on the Scottish Index of Multiple Deprivation (SIMD) (Scottish Government, 2009) at data entry and then destroyed to preserve individual anonymity. Scottish Index of Multiple Deprivation was grouped into ordinal quintiles of deprivation. Age was grouped into two-year categories (11–12 up to 17–18) corresponding approximately to school years. For individuals missing age data, we imputed a value based on their school year.

Data were analysed using Intercooled STATA version 9.2 (STATA Corp Ltd, College Station, TX, USA) and R version 2.9.0 (R project for statistical computing, open-source, free software). The institutional and student response rates by type of educational establishment were determined and the characteristics of the sample described. A comparison was made between the characteristics of the total sample and the 1866 samples included in the final analysis using Fisher's exact and *χ*^2^-tests. Prevalence estimates and 95% CIs were calculated. A crude and weighted stratified analysis of HPV prevalence by sex was conducted using the survey package in R, with schools nested within EAs. Sample weights were calculated to account for the sampling proportional to size of the EA and response bias. We compared the prevalence of HPV in 11- to 14-year olds (which includes those aged 12–13 years, the target of the primary vaccination programme) with the prevalence in 15- to 18-year olds (which includes those targeted as part of the catch-up immunisation campaign). The percentage of infections and 95% CIs due to infections with the bivalent vaccine types 16 and 18, with high-risk types and with multiple types were calculated as ratio estimates for females aged 15–18 years. Weighted odds ratios (OR) for HPV infection were calculated using logistic regression accounting for the survey design. Two separate regressions were carried out, one restricted to females attending schools and another on all females aged 16–18 years (including those attending college), as there is confounding between age, attending a college and deprivation. No regression analysis was carried out using the data for males and females together, as the data for males were inconsistent and did not exhibit the same trends. All estimates were weighted to account for the age-stratified sampling strategy and to adjust for the lower response rate associated with high deprivation. Weights were calculated on the basis of the mid-year population estimates for Scotland (General Register Office for Scotland, 2008). Only those who provided an adequate sample for HPV detection were included in the final analyses.

## Results

### Response to the study

Of the 32 EAs in Scotland, 8 were invited and all agreed to participate in the study; 51% (58/114) of state schools, 75% (3/4) of independent schools and 60% (9/15) of colleges agreed to take part. Within schools, the response rate, calculated on the basis of the number of registered students in a year and not on the number who actually attended the study presentation, was estimated at 15% (2219/14 417) overall. The response rate was 14% (1980/13 713) for state schools (range, 0.3–45% per individual school) and 34% (239/704) for independent schools (range, 18–39%). The response rate at colleges was not recorded, as reliable denominator data was not available.

### Sample characteristics

Excluding leaking samples and those with insufficient volume, 2447 (95%) of 2575 submitted samples were suitable for processing; 1866 (72%) contained amplifiable DNA and were classified as valid for HPV detection. Submission of an invalid sample was not related to age, deprivation, recruitment from an urban or rural setting or attending a denominational school (Table 1). Of the invalid samples (without amplifiable DNA), 75% came from males; males were twice as likely as females to submit invalid samples. Following the exclusion of these samples, the proportion of males in our sample decreased from 47 to 39%, and was significantly lower than that for females (*P*<0.001).

In the general Scottish population of 11- to 18-year olds, ∼21% are from areas of high deprivation (most deprived quintile; General Register Office for Scotland, 2008); 15% (280) of the 1866 participants in the final analysis came from areas of high deprivation (Table 1). A total of 10% (189) were aged 11–12 years; 17% (309) 13–14 years; 42% (774) 15–16 years and 32% (594) 17–18 years. In total, 9% (174) attended independent schools (compared with ∼5.5% of the Scottish secondary school population (HM Inspectorate of Education, 2006)) and 12% (228) attended colleges. In Scotland, ∼55% of 17-year olds are still in school (HM Inspectorate of Education, 2006), compared with 72% of our sample of 17- to 18-year olds. Approximately 11% (213) of respondents attended denominational schools, compared with 17% nationally (HM Inspectorate of Education, 2006).

### HPV prevalence among females

Among females aged 11–14 years (Table 2), the weighted prevalence of HPV was 1.1% (95% CI: 0.0–2.8). No 11- to 14-year-old females were infected with the bivalent vaccine types 16 and 18. The weighted prevalence of HPV-6 and -11 was 0.1% (95% CI: 0.0–0.4). The weighted prevalence of high-risk infections (infections with types 16, 18, 26, 31, 33, 35, 39, 51, 53, 56, 58, 59, 66, 68, 69, 70, 73 and 82) was 0.9% (95% CI: 0.0–2.6). None had multiple infections.

**Figure 1:**
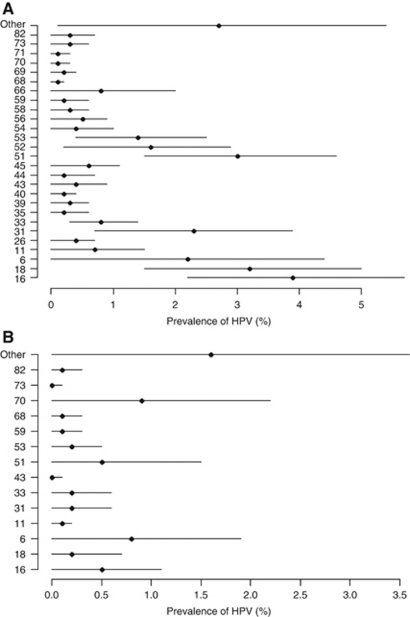
Type-specific prevalence of HPV infection among (**A**) female and (**B**) male students aged 15–18 years in Scotland in 2008.

Among the 15- to 18-year olds, the weighted prevalence was 15.2% (95% CI: 10.8–19.7); 42.5% (95% CI: 32.7–52.2) of all infections were with types 16 and 18 (weighted prevalence=6.5% 95% CI: 4.3–8.6). The weighted prevalence of HPV-6 and -11 was 2.9% (95% CI: 0.7–5.1). High-risk infections accounted for 82.7% (95% CI: 76.0–89.3) of all infections (weighted prevalence=12.6% 95% CI: 9.3–15.9); 50.3% (95% CI: 40.9–59.6) had multiple infections (weighted prevalence=7.7% 95% CI: 4.6–10.7). Human papillomavirus 16 was most prevalent (weighted prevalence=3.9% 95% CI: 2.2–5.7; Figure 1), followed by HPV-18 (weighted prevalence=3.2% 95% CI: 1.5–5.0), HPV-51 (weighted prevalence=3.0% 95% CI: 1.5–4.6), HPV-31 (weighted prevalence=2.3% 95% CI: 0.7–3.9) and HPV-6 (weighted prevalence=2.2% 95% CI: 0.0–4.4).

### Independent risk factors for HPV infection

Among females attending school (Table 3), age was a statistically significant predictor of HPV positivity (*P*-trend=0.004), with a much higher prevalence among those aged 17–18 years.

There was a trend (*P*=0.017) towards higher positivity with deprivation, although this trend was not strictly linear. In comparison to females in the most affluent quintile, those in the medium quintile of deprivation had a greater OR of being HPV positive (OR=2.4; 95% CI: 0.9–6.5) than those in the second most deprived quintile (OR=1.4; 95% CI: 0.7–2.6). Overall, compared with those in the most affluent quintile, the OR of being HPV positive was greatest among those in the most deprived quintile (OR=3.4; 95% CI: 1.0–12.0).

There was no evidence that HPV positivity varied according to state versus independent school, school denomination or urban versus rural setting of the school.

Among females aged 16–18 years, those aged 17–18 years had increased odds of being positive (of 1.9; 95% CI: 1.0–3.6) compared with those aged 16 years. There was also a statistically significant trend (*P*-trend=0.045) towards increased risk of infection with increasing deprivation. Again this trend was not linear, as the risk of infection was highest in those from the second most deprived quintile (OR=3.0; 95% CI: 1.3–7.1) rather than in those from the most deprived quintile (OR=2.1; 95% CI: 0.7–6.5). A sizeable proportion of 16- to 18-year olds (21% of the sample) attend colleges. We observed an association between HPV infection and attending a college rather than an urban school (OR=5.4; 95% CI: 3.5–8.4). All colleges are in urban areas. Adjusting for deprivation, females attending rural schools were more likely to be infected with HPV than females in urban schools (OR=2.2; 95% CI: 1.4–3.6). Again, denominational affiliation of the school had no association with HPV status.

### HPV prevalence among males

Among males aged 11–14 years (Table 2), the weighted prevalence of HPV was 1.4% (95% CI: 0.0–2.9). The weighted prevalence of types 16 and 18 was 0.7% (95% CI: 0.0–1.5). The weighted prevalence of HPV-6 and -11 was 0.7% (95% CI: 0.0–1.8). The weighted prevalence of high-risk infections was 1.0% (95% CI: 0.0–2.1) and of multiple infections was 0.3% (95% CI: 0.0–0.9).

Among males aged 15–18 years, the weighted prevalence was 3.9% (95% CI: 1.3–6.6). The weighted prevalence of types 16 and 18 was 0.7% (95% CI: 0.0–1.5). The weighted prevalence of HPV-6 and -11 was 0.9% (95% CI: 0.0–1.9). The weighted prevalence of high-risk infections was 2.4% (95% CI: 0.0–4.7) and of multiple infections was 1.2% (95% CI: 0.0–2.4). Human papillomavirus 70 was most prevalent (weighted prevalence=0.9% 95% CI: 0.0–2.2; Figure 1), followed by HPV-6 (weighted prevalence=0.8% 95% CI: 0.0–1.9), HPV-16 (weighted prevalence=0.5% 95% CI: 0.0–1.1) and HPV-51 (weighted prevalence=0.5% 95% CI: 0.0–1.5).

## Discussion

This study provides information on the overall and type-specific prevalence of HPV infection by age, sex and social deprivation, and on the distribution of vaccine and non-vaccine types and high- and low-risk types in a sample of the general unvaccinated adolescent population. Few direct population-based studies of HPV have been conducted, few have recruited participants in the age range targeted for vaccination (⩽13 years) and few have included males.

### Female prevalence

In our sample of 11- to 14-year olds, the overall weighted prevalence of HPV was low (1%); no infections were associated with the bivalent vaccine types 16 and 18, although one infection with HPV type 6 (which can be prevented by the quadrivalent vaccine) was detected. In this age group, most of those who were infected were infected with a high-risk type. Among females aged 15–18 years, the overall weighted prevalence of HPV was substantially higher (15%); over 80% were infected with high-risk types and almost half of all infections were with types 16 and 18.

There is some variation in the HPV prevalence estimates reported in surveys of the general population of adolescent women, and as most of these have used cervical swabs or brush samples, which have a higher sensitivity than urine, a direct comparison with our results is difficult. Nonetheless, a survey of 15- to 17-year-old female secondary school students in Sweden (Andersson-Ellstrom *et al*, 1996) reported a similar HPV prevalence rate of 12%, and, although a survey of 14- to 19-year-old females in the United States (Dunne *et al*, 2007) reported a higher prevalence rate of 25%, we detected a similar prevalence of 24% among our 17- to 18-year-old respondents. The prevalence rate among the 17- to 18-year-old women in our study is also comparable with that reported among 15- to 19-year olds attending cervical screening in England (Peto *et al*, 2004). To our knowledge, only a couple of other surveys have used urine as a sample for HPV detection; these have targeted older women (aged 18–25 years) who were selected on the basis of either being sexually inexperienced (prevalence rate=6% Prustya *et al*, 2005) or sexually active (prevalence rate=27% Manhart *et al*, 2006), making a direct comparison with our results inappropriate.

In our sample, increasing age, and among the older age groups, increasing deprivation, attending college and attending a rural school were associated with HPV infection. The association between HPV infection and deprivation was not absolute, as the risk of infection did not increase in a completely linear manner with deprivation. Overall though, those in more deprived areas were more at risk of infection than those in the most affluent areas. Attending a college was more strongly associated with HPV infection than social deprivation; in our sample, 40% of those attending college resided in the most deprived areas (highest quintile of deprivation, as defined by the SIMD). This is important because it is recognised that those from deprived areas are less likely to attend cervical screening (NHS Greater Glasgow and Clyde, 2006) and more likely to develop cervical cancer (NHS Information Services Division, 2007).

### Male prevalence

The HPV prevalence rates detected for males in both age groups was low and is lower than rates reported in most other studies (Dunne *et al*, 2006). Among males, the prevalence of HPV was almost threefold lower among 11- to 14-year olds compared with 15- to 18-year olds. The prevalence of vaccine types was the same, and a similar prevalence of HPV types 6 and 11 (the most common types associated with genital warts) were observed.

Males aged 11–14 years had a similar prevalence of HPV compared with females of the same age, although the prevalence of types 16, 18, 6 and 11 was higher. Conversely, males aged 15–18 years had an almost fourfold lower prevalence than that reported among females of the same age. The difference in prevalence by sex in this older age group may be explained by a number of factors, in particular, by the much lower sensitivity of the urine assay in males and by the shorter duration of infection in males (Giuliano *et al*, 2008) compared with females (Ho *et al*, 1998; Woodman *et al*, 2001; Richardson *et al*, 2003). Males aged 14–19 years have been reported to have a seroprevalence of over ninefold lower than females of the same age (Dunne *et al*, 2009). Adolescent males also tend to have younger female partners who may have lower rates of infection. A similar age-related discrepancy in rates of Chlamydia infection has previously been reported (Fenton *et al*, 2001).

The pattern of HPV infection among the general population of young adolescent males requires further investigation. Additional research is warranted to quantify the impact of female vaccination on herd immunity in males, although a reduction in the incidence of genital warts in heterosexual males of an age similar to that of vaccinated females has already been demonstrated (Fairley *et al*, 2009). This information will allow the cost effectiveness of HPV immunisation programmes to be quantified more accurately.

### Limitations

Our study was subject to a number of limitations, which are likely to have resulted in an underestimation of HPV prevalence in the study population.

We used urine as a sample for HPV detection in this survey. The sensitivity and specificity of urine for HPV detection is lower than other sample types such as genital swabs. The lower sensitivity (55.9%) and specificity (63.2%) of the assay in male samples compared with female samples (90.5 and 71%, respectively), as determined from the assay validation study, would have led to the systematic underdetection of HPV infection among males. In addition, samples from males in our study were twice as likely as samples from females to contain no amplifiable DNA, as determined by the absence of the cellular housekeeping gene, and therefore to be invalid for HPV detection. Given this, it is likely that we have underestimated the prevalence of HPV, particularly in our sample of males, although we expect the distribution of HPV types to be accurate, as the assay validation study demonstrated a similar distribution of HPV types in urine compared with gold standard specimens.

Those assumed to be most at risk of HPV infection were underrepresented in our sample because of the lower response rate from those from areas of high social deprivation, in which indicators of sexual health are known to be poorer (Scottish Executive, 2006), and also because the study design excluded those 11- to 18-year olds who were no longer in education and training.

We estimated the response rate to the study (based on the number of registered students in the school years and not on the number of students who actually attended the study presentations) to be ∼14%. Although this is likely to be an underestimate, the overall response was still low. This was, in part, due to the low acceptability of donating a urine sample in this population. Similar problems have previously been encountered by other population-based surveys of HPV, particularly those employing genital swabs, with response rates as low as 4% being reported in recent literature (Partridge *et al*, 2007).

## Conclusion

HPV prevalence is low in the 11–14 year age group, which includes the age group targeted for routine vaccination and so supports previous evidence used to recommend the optimum age to offer the vaccine. The relationship between deprivation and HPV prevalence requires further investigation, especially in relation to the need to ensure a high uptake of HPV immunisation among those who are less likely to attend cervical screening. The picture of HPV infection among males is unclear and requires further investigation.

## Figures and Tables

**Table 1:** Characteristics of respondents to a survey of HPV infection among students in schools and colleges in Scotland from January to May 2008

	**All respondents (*N*=2575), % (*n*)**	**Respondents submitting valid samples (*N*=1866), % (*n*)**	***P*-value**
*Sex*			
Male	46.2 (1190)	38.8 (724)	*P*<0.001
Female	52.1 (1341)	60.1 (1121)	
Not reported	1.7 (44)	1.1 (21)	
			
*Age group (years)*			
11–12	10.7 (276)	10.1 (189)	*P*=0.459
13–14	16.5 (424)	16.6 (309)	
15–16	39.2 (1009)	41.5 (774)	
17–18	33.6 (866)	31.8 (594)	
			
*Deprivation category* a			
Low	24.0 (619)	23.8 (444)	*P*=0.772
Medium–low	21.4 (551)	21.2 (396)	
Medium	17.7 (457)	17.6 (329)	
High–medium	17.7 (457)	17.8 (333)	
High	14.8 (381)	15.0 (280)	
Not reported	4.3 (110)	3.4 (63)	
			
*Type of educational institute*			
Independent school	8.5 (209)	9.3 (174)	*P*=0.005
State school	78.3 (1915)	78.5 (1464)	
College	13.2 (323)	12.2 (228)	
			
*Denominational school*			
No	89.2 (2297)	88.6 (1653)	*P*=0.197
Yes	10.8 (278)	11.4 (213)	
			
*Setting*			
Rural	27.7 (714)	28.4 (530)	*P*=0.636
Urban	72.3 (1860)	71.6 (1336)	

Abbreviation: HPV=human papillomavirus.

a Deprivation category is derived from Scottish Index of Multiple Deprivation.

**Table 2:** Weighted HPV prevalence among male and female students aged 11–14 and 15–18 years in Scotland in 2008 (*n*=1866)

**HPV type**	**Females aged 11–14 years, % (95% CI)**	**Females aged 15–18 years, % (95% CI)**	**Males aged 11–14 years, % (95% CI)**	**Male aged 15–18 years, % (95% CI)**
All-types	1.1 (0–2.8)	15.2 (10.8–19.7)	1.4 (0.0–2.9)	3.9 (1.3–6.6)
HPV-16 and -18 (bivalent vaccine types)	0.0 (0.0–0.0)	6.5 (4.3–8.6)	0.7 (0.0–1.5)	0.7 (0.0–1.5)
Non-vaccine types (types other than 16 and 18)	1.1 (0–2.8)	8.8 (5.5–12.0)	0.7 (0.0–1.6)	3.2 (0.9–5.6)
Non-vaccine types 6 and 11	0.1 (0–0.4)	2.9 (0.7–5.1)	0.7 (0.0–1.8)	0.9 (0.0–1.9)
High-risk types (single or multiple infections±low-risk types)^a^	0.9 (0–2.6)	12.6 (9.3–15.9)	1.0 (0.0–2.1)	2.4 (0.0–4.7)
Low-risk types only (multiple and single infections)^b^	0.1 (0–0.4)	2.6 (1.1–4.2)	0.4 (0.0–1.0)	1.6 (0.0–3.4)
Multiple infections	0.0 (0.0–0.0)	7.7 (4.6–10.7)	0.3 (0.0–0.9)	1.2 (0.0–2.4)
				
*HPV infections*				
Single type	1.1 (0.0–2.8)	7.6 (5.4–9.8)	1.1 (0.0–2.3)	2.8 (0.6–5.0)
Two types	0.0 (0.0–0.0)	4.3 (1.5–7)	0.0 (0.0–0.0)	0.8 (0.0–1.9)
Three types	0.0 (0.0–0.0)	2.6 (1.2–4.0)	0.3 (0.0–0.9)	0.2 (0.0–0.6)
Four types or more	0.0 (0.0–0.0)	0.8 (0.0–1.6)	0.0 (0.0–0.0)	0.2 (0.0–0.4)

Abbreviation: HPV=human papillomavirus.

a High-risk types include types 16, 18, 26, 31, 33, 35, 39, 51, 53, 56, 58, 59, 66, 68, 69, 70, 73 and 82.

b Low-risk types include types 6, 11, 40, 43, 44, 54 and 71.

**Table 3:** Factors associated with HPV infection among (model A) females attending secondary schools and (model B) all 16- to 18-year-old females in Scotland in 2008

**Variable**	**% (95% CI)**	**Odds ratio (95% CI)**	***P*-value**
*Model A: females in school*			
Age group (years)			
11–12	0.4 (0.0–1.3)	0.1 (0.0–0.8)	0.004^a^
13–14	1.4 (0.0–4.1)	0.2 (0.0–1.8)	
15–16 (ref)	6.9 (3.8–10.0)	1	
17–18	15.8 (11.1–20.4)	2.5 (1.4–4.5)	
Deprivation category			
Low (ref)	3.4 (1.4–5.5)	1	
Medium–low	3.6 (1.3–6.0)	1.0 (0.4–2.6)	0.017^a^
Medium	9.2 (2.8–15.7)	2.4 (0.9–6.5)	
High–medium	4.5 (1.8–7.2)	1.4 (0.7–2.6)	
High	7.7 (0.0–16.5)	3.4 (1.0–12.0)	
Type of educational institute			
Independent school (ref)	2.5 (0.0–5.7)	1	0.829
State school	5.9 (3.5–8.3)	1.2 (0.3–4.6)	
Denominational school			
No (ref)	5.5 (3.1–7.8)	1	0.312
Yes	6.9 (0.0–14.4)	0.6 (0.2–1.7)	
Setting			
Rural (ref)	5.7 (1.6–9.7)	1	0.734
Urban	5.5 (3.0–8.1)	0.9 (0.4–1.9)	
			
*Model B: females aged 16–18 years*			
Age group			
16 (ref)	11.1 (4.0–18.2)	1	0.071
17–18	24.2 (16.9–31.5)	1.9 (1.0–3.6)	
Deprivation Category			
Low (ref)	9.9 (4.3–15.6)	1	0.045^a^
Medium–low	9.9 (2.7–17.1)	0.9 (0.3–2.8)	
Medium	17.9 (9.2–26.6)	1.6 (0.8–3.3)	
High–medium	27.7 (14.2–41.3)	3.0 (1.3–7.1)	
High	24.9 (12.0–37.8)	2.1 (0.7–6.5)	
Type of educational institute			
Urban school (ref)	9.2 (5.0–13.5)	1	<0.001
Rural school	14.4 (8.6–20.2)	2.2 (1.4–3.6)	
Urban college	38.3 (31.1–45.5)	5.4 (3.5–8.4)	
Denominational school			
No (ref)	19.8 (13.8–25.7)	1	0.700
Yes	13.0 (0.0–25.9)	1.2 (0.5–2.9)	

Abbreviations: HPV=human papillomavirus; ref=reference value.

a *P*-value for trend.

## Appendix 1

### Laboratory analysis

#### Specimen transit, processing and extraction

Urine was transported to the laboratory and refrigerated, ideally within 24 h of collection, at 4°C, for a maximum of 7 days, then stored at −70°C. Urine samples were thawed in batches, a 5-ml aliquot was removed and centrifuged at 2800 r.p.m. for 20 min and the supernatant was discarded; 3 ml of sterile phosphate-buffered saline (PBS) was added to the pellet, vortexed until fully re-suspended, centrifuged for a further 20 min at 800 *g*, the supernatant discarded and 1 ml of PBS added. Washed urine suspensions were frozen at −70°C, pending nucleic acid extraction. The 1 ml washed urine suspensions were extracted on an MDX (Qiagen Ltd., Crawley, UK) platform using the Qiagen Media Kit according to manufacturer's instructions (Qiagen, Crawley, UK). This method uses ∼300 μl for sample input and elutes nucleic acid in ∼100 μl.

#### HPV genotyping

HPV detection was performed using the HPV Inno-LiPA HPV Genotyping Extra assay (Innogenetics). This method involves the PCR amplification of a 65-bp region of HPV L1 DNA and can resolve 27 different HPV types; 20 high-risk types (including the 15 types classified as high-risk according to the International Agency on Research on Cancer); and 7 low-risk types (including HPV-6 and -11). The assay also incorporates a control for human DNA in order to ascertain specimen quality/cellularity and exclude ‘false-negative’ samples. All molecular work was carried out within a purpose-built, zoned molecular suite and a positive and negative control was included for every 18 samples tested.
